# The Dark Side of the Salad: *Salmonella typhimurium* Overcomes the Innate Immune Response of *Arabidopsis thaliana* and Shows an Endopathogenic Lifestyle

**DOI:** 10.1371/journal.pone.0002279

**Published:** 2008-05-28

**Authors:** Adam Schikora, Alessandro Carreri, Emmanuelle Charpentier, Heribert Hirt

**Affiliations:** 1 Unité de Recherche en Génomique Végétale, Institut National de la Recherche Agronomique/Centre National de la Recherche Scientifique/University of Evry Val d'Essonne, Evry, France; 2 Department of Plant Molecular Biology, Max F. Perutz Laboratories, Vienna, Austria; 3 Department of Microbiology and Immunobiology, Max F. Perutz Laboratories, Vienna, Austria; University of California Merced, United States of America

## Abstract

*Salmonella enterica* serovar *typhimurium* contaminated vegetables and fruits are considerable sources of human infections. Bacteria present in raw plant-derived nutrients cause salmonellosis, the world wide most spread food poisoning. This facultative endopathogen enters and replicates in host cells and actively suppresses host immune responses. Although *Salmonella* survives on plants, the underlying bacterial infection mechanisms are only poorly understood. In this report we investigated the possibility to use Arabidopsis thaliana as a genetically tractable host system to study Salmonella-plant interactions. Using green fluorescent protein (GFP) marked bacteria, we show here that *Salmonella* can infect various Arabidopsis tissues and proliferate in intracelullar cellular compartments. Salmonella infection of Arabidopsis cells can occur via intact shoot or root tissues resulting in wilting, chlorosis and eventually death of the infected organs. Arabidopsis reacts to *Salmonella* by inducing the activation of mitogen-activated protein kinase (MAPK) cascades and enhanced expression of pathogenesis related (*PR*) genes. The induction of defense responses fails in plants that are compromised in ethylene or jasmonic acid signaling or in the MKK3-MPK6 MAPK pathway. These findings demonstrate that Arabidopsis represents a true host system for *Salmonella*, offering unique possibilities to study the interaction of this human pathogen with plants at the molecular level for developing novel drug targets and addressing current safety issues in human nutrition.

## Introduction

*Salmonella enterica* serovar *typhimurium* (*S. typhimurium*) is a facultative endopathogen and the causative agent of various human diseases ranging from enteritis to typhoid fever. It is responsible for salmonellosis, which is the most frequent food-borne disease with around 1.5 billion yearly infections world wide (WHO). Disease in mammals occurs after consumption of contaminated food or water. Systemic infection can follow, which depends on the ability of the bacteria to survive the harsh conditions of the gastric tract before crossing the intestinal epithelium. Owning to its importance, the mechanism of *Salmonella* invasion of human cells is under intense study. Two *Salmonella* pathogenicity islands (SPI-1 and SPI-2) encoding structural elements of type III secretion systems (T3SS) and effectors injected into the host cells, are necessary for entry and proliferation within mammalian cells [1]. Infection occurs in several well-organized and adjusted steps. The first one includes docking of *Salmonella* to the epithelial cell and injection of SPI-1 encoded effectors, which suppress the host immune system and modify the actin and tubulin cytoskeleton [2]. Endocytosis is the second step and requires formation of Salmonella Containing Vacuoles (SCVs). Retainment of SCVs in the host cytoplasm is assured by SPI-2 encoded effectors [3] and strains not capable to sustain intact SCVs are avirulent [4].

*Salmonella*-contaminated vegetables and fruits were recently identified as a widespread source of human infection [5]. Although diverse plant species support growth of *Salmonella* [6], the underlying molecular mechanisms of the *Salmonella*–plant interaction are largely unknown.

The plant immune system functions at different levels during pathogen attack. Pathogen-associated molecular patterns (PAMPs) and effectors injected into plant cells, trigger activation of defence mechanisms [7], [8], [9], [10]. PAMPs are recognized by plant receptors and trigger a defence mechanism referred to as “basal” defence [11]. On the other hand, pathogens have developed mechanisms to overcome detection by injecting effectors into plant cells, which interfere with signalling cascades and thereby abolish basal defence response. In some cases, pathogen effectors can be recognized by plant resistance proteins (R proteins), triggering a hypersensitive response (HR) and thereby limit pathogen infection [12]. Systemic acquired resistance (SAR) represents another level of defence resulting in resistance to a broad spectrum of pathogens throughout the plant [13]. SAR requires signal molecules activating the expression of pathogenesis-related (*PR*) genes and salicylic acid (SA), jasmonic acid (JA) and ethylene (ET) are involved in regulating their expression. These molecules play important roles in the induction of plant defence responses upon pathogen attack and are implicated in different forms of resistance [14], [15]. While SA-dependent pathways are generally important in defence against biotrophic pathogens and lead to hypersensitive response (HR) and/or local resistance [13], JA and ET pathways seem to be involved in defence mechanisms against herbivore attack and necrotrophic pathogens [16].

Mitogen-activated protein kinase (MAPK) cascades play a crucial role in mediating defence responses to plant pathogen attack [17]. In *Arabidopsis*, MPK3 and MPK6 are activated by different bacterial elicitors [18], [19] and trigger enhanced expression of *PR* genes [7], [20]. Silencing of *MPK6* results in compromised resistance to plant pathogens [21]. MEKK1, MKK4/5, MPK3/6/4 were identified downstream of the FLS2 receptor recognizing bacterial flagellin [7] and are also involved in regulating reactive oxygen species (ROS) production [22], [23], [24]. Recently, MKK3 was shown to have a dual function; activating either MPK6 in response to JA signalling [25], or MPK7 in response to ROS and plant pathogens [26].

Comparing the defence response to classical plant pathogens, the response to human pathogens is much less understood. The human pathogen *S. aureus* triggers SA-dependent defence responses in *Arabidopsis* plants [27] and its spreading can be diminished by SA treatment. In addition, the SA-depleted isochorismate synthase defective *ics1* mutants and transgenic *Arabidopsis* lines, overexpressing the bacterial SA hydroxylase (*NahG*), are hypersensitive to *S. aureus* infection [27]. Recently, it was reported that treating plants with the ethylene precursor ACC, significantly diminishes colonization of *Medicago sativa* and *Arabidopsis* by *Klebsiella pneumoniae* or *Salmonella* [28].

The present study shows that the human pathogen *S. typhimurium* triggers the activation of plant immune responses including enhanced transcription of *PR* genes. We show that *Salmonella* can overcome plant defence mechanisms and enter and proliferate inside various *Arabidopsis* tissues, causing wilting and chlorosis as disease symptoms. Among different possible immune responses of *Arabidopsis*, the ET- and JA-dependent signalling pathways were found of major importance for inducing defence responses during *S. typhimurium* infection. Moreover, the JA-mediating MKK3-MPK6 MAPK pathway was identified to be essential for restricting *Salmonella* proliferation and disease in *Arabidopsis* plants. Our results indicate that *Arabidopsis can* serve as a valuable model system to study Salmonella-host interaction, suggesting the necessity to modify agricultural practices to improve food safety

## Results

### Salmonella is pathogenic to Arabidopsis

**Figure 1:**
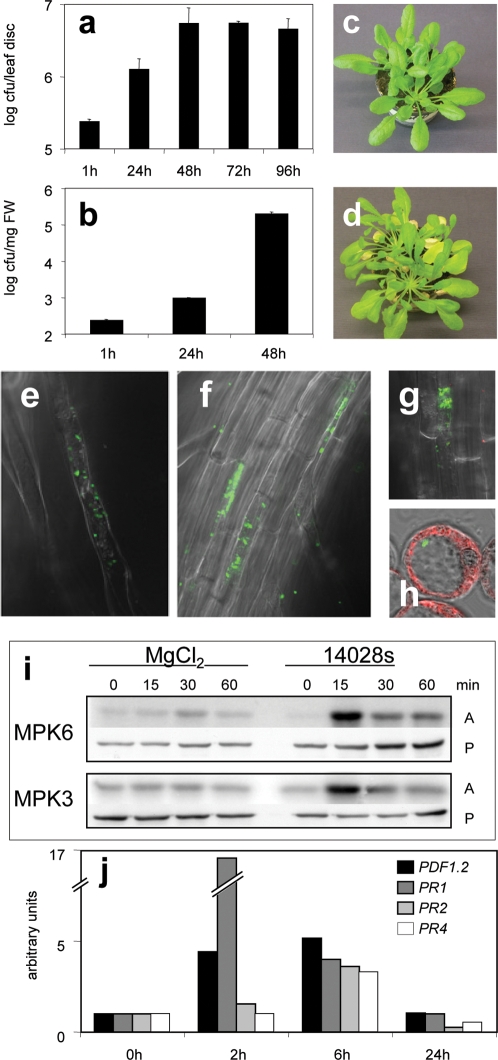
*Salmonella* can invade and grow in *Arabidopsis* cells and induce plant innate defence mechanisms. a–b, Three weeks old *A. thaliana* plants were vacuum-infiltrated (a) and 14 days old *A. thaliana* seedlings were incubated in medium inoculated (b) with *S. typhimurium* wild type 14028s. Proliferation of *Salmonella* was analyzed over a period of 4 (a) and 2 (b) days by determination the cfu of internal bacteria in homogenates of leaf discs (a) or seedlings (b). c–d; Disease symptoms after dipping in *Salmonella*. Three weeks old *A. thaliana* plants were dipped for 5 min in *S. typhimurium* 14028s solution and macroscopic changes were observed over a 2 weeks period, c: before dipping, d: 14 days after dipping in bacterial solution. e–h, *Salmonella* enters plant cells. GFP marked *S. typhimurium* 14028s were localized in *A. thaliana* root cells after 3 (e) or 20 h (f, g). h; 3 h after infection, cell culture protoplasts contain bacterial cells. Images were taken using a confocal microscope with 488 nm excitation and 505–530 nm emission filters. i, Treatment with *Salmonella* induces MPK3 and MPK6 kinase activities. Two weeks old *A. thaliana* seedlings were treated with either 10 mM MgCl_2_ or *S. typhimurium* 14028s. Endogenous MPK6 was immunoprecipitated from total protein extraction. Myelin basic protein (MBP) was used as substrate. A, activity; P, protein amounts were detected by Western blotting with antibodies specific for MPK3/6. j, Treatment with *Salmonella* induces defence responses. 14 days old *A. thaliana* seedlings were treated with *S. typhimurium* 14028s. Quantitative RT-PCR analysis was performed on 5 µg reverse transcribed total mRNA; *clathrin*, *ubiquitin4* and *actin2* were used for normalization.

Three different types of experiments were performed to assess the question whether *Salmonella* can actively invade, proliferate and spread through plants. To test whether *Salmonella* is capable to proliferate inside plants, whole rosettes of *Arabidopsis thaliana* Col-0 wild-type plants were vacuum-infiltrated with *S. typhimurium* wild-type strain 14028s and the internal bacterial population was then counted over the following four days. The number of colony forming units (cfu) in discs excised from infiltrated leaves drastically increased over the first 2 days before reaching plateau levels (Fig. 1a). Two weeks after dipping the plants in bacterial solutions, invasion of *Salmonella* into *Arabidopsis* caused a defined disease phenotype as revealed by severe chlorosis and wilting of leaves (Fig. 1c–d). To evaluate whether *Salmonella* is also able to actively recognize and invade *Arabidopsis*, liquid media in which *Arabidopsis* seedlings were immerged, were inoculated with bacteria. Over the following days, the bacterial population inside seedlings was monitored after surface-sterilization and homogenisation of the seedlings (Fig. 1b). A 40-fold increase in cfu of internal bacteria was observed over a period of 2 days (Fig. 1b). Altogether these results indicate that *Salmonella* can actively invade and proliferate in *Arabidopsis* plants and cause disease.

Most pathogenic bacteria can spread through susceptible host plants very rapidly. We investigated whether *Salmonella* can also systemically infect plants. For this purpose we selectively infected roots with *Salmonella* and analyzed its presence in leaves two weeks later. While bacteria were growing on and in Arabidopsis roots two weeks after infection, we were not able to detect *Salmonella* in leaf homogenates from these plants (data not shown). Alternatively, to determinate whether *Salmonella* can spread from leaf-to-leaf, we syringe-infiltrated single leaves and monitored the presence of bacteria in non-infiltrated neighbouring leaves two weeks later. Although *Salmonella* effectively infected single leaves, no bacteria were detected in non-infiltrated neighbouring leaves (Fig. S1). These data indicate that *S. tyiphimurium* is not able to spread to other non-exposed parts of the *Arabidopsis* plant, suggesting that *Salmonella* cannot migrate via the xylem (root-to-shoot) nor via the phloem (leaf-to-leaf) systems.

From the epidemiological standpoint, it is important to know the duration of *Salmonella* persistence in infected plants. We therefore determined how long *Salmonella* could stay alive in Arabidopsis plants. For this purpose, whole rosettes of three weeks old *Arabidopsis* plants were vacuum-infiltrated and the presence of internal bacteria was monitored over the period of one month. Although the infiltrated leaves died within 5 days, apical meristems survived the infection and produced new leaves. One month after infiltration, newly formed leaves contained significant albeit much lower *Salmonella* cfu, possibly indicating that meristems are poor *Salmonella* targets or that meristematic tissues are particularly resistant to bacterial infection (Fig. S2).

### Endopathogenic lifestyle of *Salmonella* in *Arabidopsis*

In animals and humans, *Salmonella* actively enters epithelial and other cells in order to replicate and spread through the organism. We focused our attention on the question whether, similar to the situation in mammals, *Salmonella* can also invade plant cells. For this purpose, *S. typhimurium* was transformed with a plasmid constitutively expressing green fluorescent protein (GFP) [29]. Three hours post-inoculation of liquid medium with immerged seedlings, GFP-marked *Salmonella* were localized inside root hairs (Fig. 1e) and at 20 hours post-inoculation, inside rhizodermal cells (Fig. 1f–g). At this time point, large numbers of motile bacteria were observed inside single host cells, confirming that *Salmonella* can proliferate in plant cells. To our knowledge, this is the first report of an infection of the plant cytoplasm by a human enteropathogen. *Salmonella* was also found to form biofilm-like structures on the surface of roots and leaves, preferentially colonizing regions around emerging lateral roots and wounded tissues (data not shown). To extend our *in planta* observations to the single cell level, inoculation experiments were also performed using *Arabidopsis* protoplasts. Probably due to the high sucrose concentration in the medium, infection rates in this system were low. However, protoplasts were clearly invaded by GFP-marked *Salmonella* within 3 hours (Fig. 1h). Optical Z-stacking allowed the exact positioning of the GFP-marked *Salmonella* to the cytoplasmic compartment of infected protoplasts (Fig. S3). These data demonstrate that *Salmonella* has the ability to enter and proliferate inside plant cells.

### *Arabidopsis* induces defence responses upon infection with *S. typhimurium*

**Figure 2:**
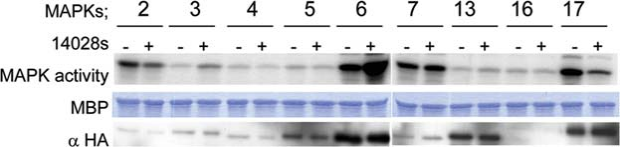
Activation of MAP kinases transiently expressed in protoplasts. MPK3 and MPK6 are activated and MPK2 and MPK17 are inhibited upon infection with *Salmonella*. Nine of 20 MAPKs were expressed in cell culture derived protoplasts for 24 h and protoplasts were exposed to bacteria for 20 min. All MAPKs were expressed as HA-tagged versions and immunoprecipitated for 2 h with 2 µl HA specific antibody and 25 µl protein A-Sepharose beads. Kinase activity assays were performed as described above.

**Figure 3:**
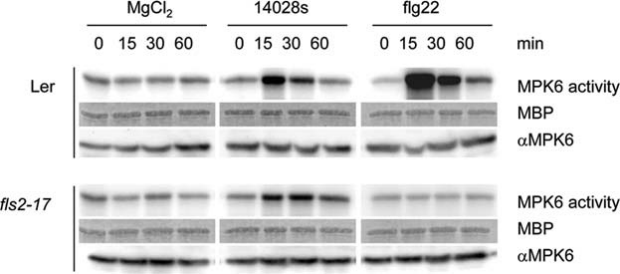
*Arabidopsis* responds to multiple *Salmonella*-derived PAMPs. Plants can recognize several *Salmonella*-derived molecules, and this recognition leads to defence mechanisms. We tested whether *Arabidopsis*, in order to activate its response to *Salmonella*, relies on the most prominent flagellum and/or other bacterial effectors. The native MPK6 can be activated by *S. typhimurium* wild type 14028s in *A. thaliana fls2–17* mutant, which is not able to recognize the flagellar protein flagellin. This result shows that the interaction between plants and *Salmonella* involves other signalling molecules, probably lipopolysaccharide (LPS), lipoproteins or other effectors. Kinase activity assays were performed on equal amounts of total protein extracted from 14 days old plants immunoprecipitated with MPK6 specific antibody. The *A. thaliana* wild type Ler was used as control in the experiment with the *A. thaliana fls2–17* mutant. Flg22 is a 22 amino-acid peptide derived from flagellin.

Under normal conditions, plants react to pathogens by activating various defence responses, thereby diminishing the proliferation of bacteria, fungi or viruses. We tested whether plants recognize *Salmonella* as a pathogen and actively induce defence responses in order to limit infection, as observed with other bacterial plant pathogens [30]. In *Arabidopsis*, a variety of PAMPs were shown to activate the MAPKs: MPK3 and MPK6 [18], [19], followed by the transcription of a number of *PR* genes [7], [20]. To determine whether these defence responses also occur upon *S. typhimurium* infection, we used MPK3 and MPK6 specific antibodies to analyze the activation of these kinases in seedlings exposed to *Salmonella*. The activities of both MPK3 and MPK6 strongly increased at 15 min after contact with the bacteria (Fig. 1i). To examine whether recognition of *Salmonella* results in activation of other MAPKs, the activities of 9 MAPKs, representing all 4 phylogenetic groups of the MAPK family in *Arabidopsis* [31], were analyzed after infection of protoplasts with *S. typhimurium*. From the nine investigated kinases, only MPK3 and MPK6 showed activation, confirming our previous *in planta* results. Activities of two other MAPKs; MPK2 and MPK17 were reduced (Fig. 2). Interestingly, *Arabidopsis* appeared to sense multiple *Salmonella* PAMPs, because *S. typhimurium* 14028s infection of an *A. thaliana fls2–17* mutant, which is defective in flagellin perception [20], still resulted in MPK6 activation (Fig. 3).

**Figure 4:**
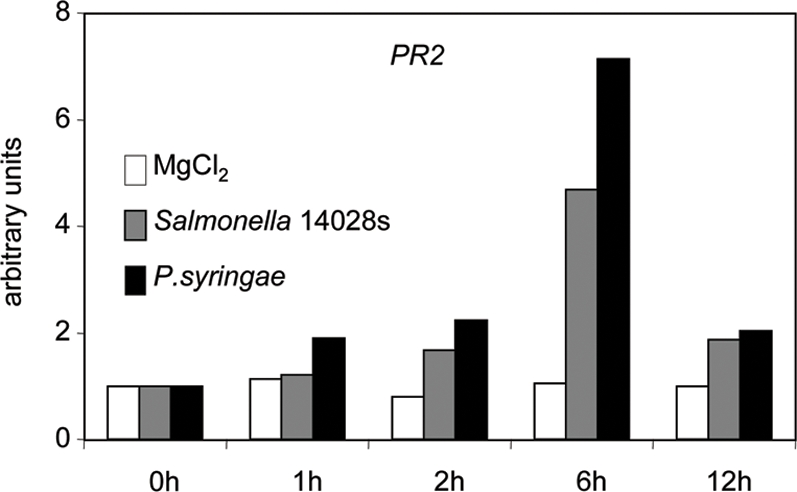
Quantitative RT-PCR analysis of JA-inducible *PR2* expression upon infection with *Salmonella* and *Pseudomonas syringae*. Transcript amounts of *PR2* (*pathogenesis related2*), similarly to *PDF1.2*, are induced by MeJA treatment. Transcript levels accumulate also after contact with *Salmonella*, pointing to a central role of the JA pathway in response to this bacterium. Moreover, the induction follows the kinetics of induction caused by the plant specialized pathogen *P. syringae*. Q-RT-PCR was performed as described above.

To study the role of the plant innate immune system during *Salmonella* infection in more detail, we turned our attention to the SA-, JA-, and ET-dependent signalling pathways. To clarify their role during infection of *Arabidopsis* with *Salmonella*, quantitative RT-PCR analysis of marker genes for SA-, JA- and ET-dependent defence gene expression was performed. *PDF1.2*, encoding an antifungal defensin, is commonly used as a marker for JA- and ET-induced resistance [32]. In *A. thaliana* Col-0 infected with *Salmonella*, expression of the *PDF1.2* gene increased rapidly during the first 6 h (Fig. 1j). Expression of other JA-regulated genes, such as *PR2* and *PR4* [33] as well as the SA marker *PR1* gene were also induced during *Salmonella* infection (Fig. 1j, Fig. 4). These results show that *Arabidopsis* reacts to *Salmonella* infection by activating multiple defence pathways of the innate immune system.

### JA- and ET-dependent signalling pathways are involved in resistance against *S. typhimurium*

**Figure 5:**
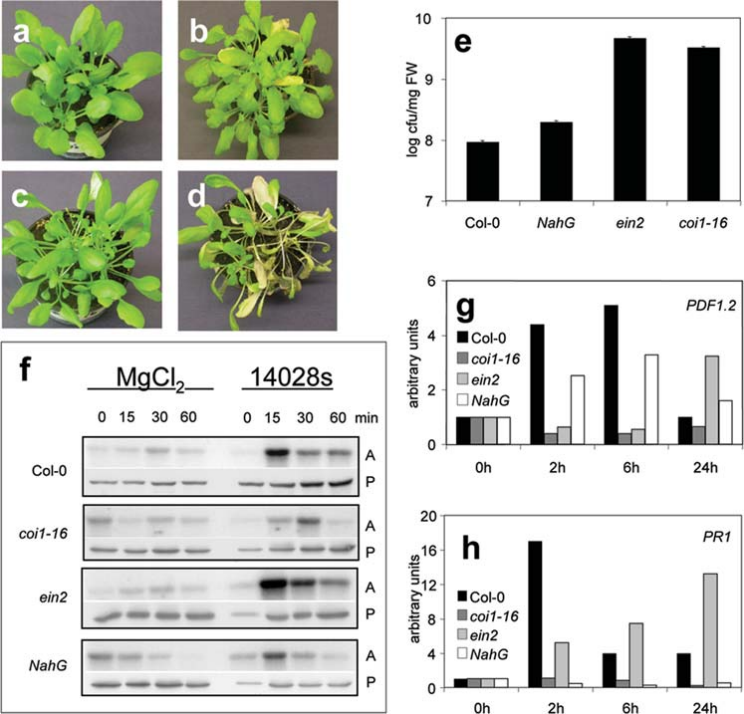
Jasmonate and ethylene signalling pathways are necessary for defence response to *Salmonella*. a–d, Disease symptoms in JA and ET insensitive mutants *ein2* (a,b) and *coi1–16* (c,d) plants dipped for 5 min in *S. typhimurium* 14028s solution; (a, c) before dipping, (b, d) 14 days after dipping.e, *coi1–16* and *ein2* are susceptible to *Salmonella* invasion. 14 days old *A. thaliana* wild type Col-0, *coi1–16* and *ein2* mutants or *NahG* transgenic seedlings were inoculated with *S. typhimurium* 14028s for 48 h, and cfu of internal bacteria in the seedling homogenates were determined. f, Activities of native MPK6. MPK6 activity was poorly induced in *coi1–16* plants after *Salmonella* treatment. Kinase activity assays were performed as described above. g–h, Quantitative analysis of JA-regulated *PDF1.2* (g) and SA-dependent *PR1* (h) expression upon *Salmonella* infection. Induction of *PDF1.2* expression was abolished in *coi1–16* and delayed in *ein2*. Expression of *PR1* was not induced in *coi1–16* or *NahG* plants. Quantitative RT-PCR analysis was performed on 5 µg reverse transcribed total mRNA. *clathrin*, *ubiquitin4* and *actin2* were used for normalization.

It was previously shown that lack of SA promotes and that treatment with the ethylene precursor ACC lowers *Salmonella* colonization of plants [28]. Therefore, we investigated whether defects in any of the three signalling pathways affect *Salmonella*'s ability to infect *Arabidopsis* plants. We performed infection experiments with SA depleted *NahG* plants [34], *ein2* (*ethylene insensitive 2)* [35] and *coi1* (*coronatine insensitive 1)* [36], [37] mutant plants compromised in either the ET or JA signalling pathways, respectively. Liquid media with immerged seedlings were inoculated with *S. typhimurium* for 48 hours before determination of cfu of the internal bacteria. When compared with wild type *A. thaliana* Col*-*0 plants, *coi1–16 and ein2-1* plants contained 35 and 50 times more *Salmonella*, respectively (Fig. 5e). In contrast, only a small difference in *Salmonella* infection levels was detected between wild type and *NahG* plants (Fig. 5e), indicating that the JA and ET pathways are important for resistance against *S. typhimurium*. Comparing the disease symptoms in *coi1–16*, *ein2-1* and *NahG* plants challenged with *Salmonella* by dipping plants for 5 min into a bacterial solution, the JA-signalling pathway appeared to be of primary importance for *Arabidopsis* resistance to *Salmonella* infection. *coi1–16* plants revealed severe senescence symptoms and wilting two weeks after dipping (Fig. 5c–d). Although *ein2-1* plants showed even higher proliferation levels of *Salmonella* than *coi1–16* plants (Fig. 5e), these plants did not shown enhanced disease symptoms (Fig. 5a–b). In contrast, *NahG* plants showed similar *Salmonella* proliferation rates and symptoms as Col-0 wild type plants (data not shown).

To better understand the different signalling events, we monitored native MPK6 kinase activities in *coi1–16*, *ein2-1* and *NahG* plants upon *Salmonella* exposure. The activation pattern of MPK6 in *ein2-1* plants was very similar to that observed in wild type *A. thaliana* Col-0 plants and strong transient MPK6 activation was observed at 15 min after bacterial contact (Fig. 5f). *NahG* plants also showed a similar activation pattern of MPK6, although total activities were generally lower (Fig. 5f). In contrast, the level of MPK6 activation was poorly above background and the activation peak was shifted towards 30 min in *coi1–16* plants (Fig. 5f).

To monitor another level of the *Arabidopsis* innate immune system, the *coi1–16*, *ein2-1* and *NahG* plants were also analysed for the expression of different defence response marker genes upon *Salmonella* treatment. In comparison to wild type *Arabidopsis* plants, *coi1–16* mutants showed no enhanced accumulation of *PDF1.2* and *PR1* transcripts (Fig. 5g–h). In *ein2-1* mutants, *PDF1.2* and *PR1* transcript accumulation was delayed upon *Salmonella* infection (Fig 5g–h). *Salmonella* treatment of *NahG* plants resulted in accumulation of *PDF1* transcripts, but no induction of *PR1* expression was detected (Fig. 5g–h). These results reveal that the Arabidopsis SA, ET and JA pathways differentially contribute to disease development and the induction of defence responses upon *Salmonella* infection.

### The MKK3-MPK6 cascade plays a crucial role in resistance to *Salmonella*

**Figure 6:**
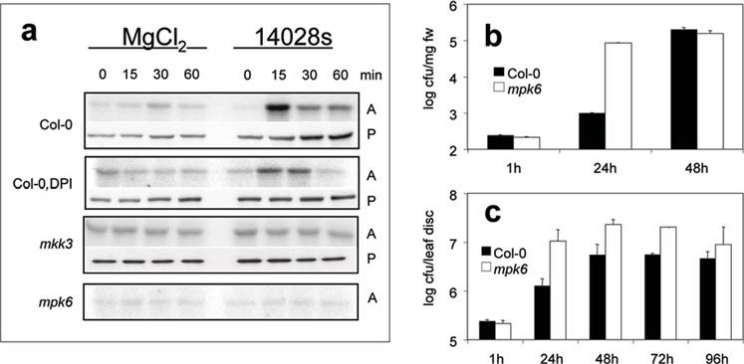
*Salmonella* induction of MPK6 depends on MKK3. a, Kinase activity assays on native MPK6 immunoprecipitated from *mkk3* and *mpk6* mutants treated with 10 mM MgCl_2_ or *S. typhimurium* 14028s. Myelin basic protein (MBP) was used as substrate. A, activity; P, protein amounts were detected by Western blotting with antibodies specific for MPK6. b, Infection assays. 14 days old seedlings incubated in MS/2 medium inoculated with *S. typhimurium* 14028s during 2 days, cfu of internal bacteria were determined from plant homogenates. c, Proliferation of bacteria in infiltrated plants is faster in *mpk6* mutants. A *S. typhimurium* 14028s solution was used to vacuum-infiltrate 3 weeks old leaves of *mpk6* or Col-0, cfu of internal bacteria were determined in homogenates of leaf discs.

Of the *Arabidopsis* mutants, *coi1–16* was compromised in MPK6 activation, all defence responses and moreover showed enhanced disease symptoms (Fig. 5). These results identify the JA signalling pathway to be of outstanding importance in *Salmonella-Arabidopsis* interaction. COI1 is an F-box protein acting in an E3 ubiquitin ligase containing complex, which degrades the repressors of JA-responsive genes [38], [39]. The JA-dependent activation of MPK6 is consistent with recent data, suggesting that full activation of MPK6 by JA requires mitogen-activated protein kinase kinase 3 (MKK3) [25]. To test this model during Arabidopsis-Salmonella interaction, we analyzed the *Salmonella*-induced activation of native MPK6 in *mkk3* mutants (Fig. 6a). Like in *mpk6* knock-out plants, no activation of MPK6 was detected in *mkk3* mutants, revealing that *Salmonella* induces MPK6 by MKK3 activation. A corollary of these results suggests that MPK6 might play an important role in defence against *Salmonella* infection. In agreement with this hypothesis, *mpk6* plants were significantly more susceptible to *Salmonella* infection, showing a 17-fold higher cfu after 24 hours of culture in liquid medium inoculated with *Salmonella* (Fig. 6b). Moreover, *mpk6* plants showed enhanced *Salmonella* proliferation levels when the bacteria were vacuum-infiltrated into leaves (Fig. 6c). These data indicate that the MKK3/MPK6 module of the JA signalling pathway plays an important role in restricting *Salmonella* infection in *Arabidopsis*.

## Discussion

The results presented in this report demonstrate that the human pathogen *Salmonella enterica* serovar *typhimurium* (*S. typhimurium*) may also be a true plant endopathogen. This bacterium can actively invade various tissues, enter into and proliferate in cells of *Arabidopsis*. Although infection of *Arabidopsis* triggers immune responses similar to those known from other plant pathogens, *S. typhimurium* is able to overcome the host defence mechanisms and multiply in *Arabidopsis* plants. Using a panel of plant defence mutants, the JA signalling pathway was identified to be of major importance for resistance of *Arabidopsis* plants to *Salmonella* infection. Overall, these results demonstrate that *Arabidopsis* can be used as a valuable genetic host model system to study *Salmonella* pathogenesis in plants.

### Salmonella is a plant endopathogen

Infection of animals or humans with *S. typhimurium* is dependent on various factors. The ability of *S. typhimurium* to enter mammalian host epithelial cells is tightly controlled by SPI-1 encoded factors and results in the formation of *Salmonella* containing vacuoles (SCVs), which are necessary for survival and subsequent systemic bacterial infection [4] [1]. The survival of *S. typhimurium* outside of the host organism is less well understood, but *Salmonella* can clearly adapt to different external conditions including low pH or high temperature [40]. *Salmonella* was not only shown to persist in soil for as long as 900 days after inoculation [41], but these bacteria can also survive in a variety of fruits, explaining why mangos and tomatoes are often causally linked to human salmonellosis. Although in these cases, food contamination is rather thought to occur during post-harvest processing, a number of recent reports indicated soil-grown vegetables as the source of human *Salmonella* infection [5]. The presence of *Salmonella* in soil might mostly originate from animal-derived contamination such as organic fertilizers or slaughter waste. The question arising now is whether infection of plants by *Salmonella* in the soil occasionally occurs via passive and nonselective mechanisms or whether infection underlies active bacterial infection processes employed by *Salmonella*. Our results, as well as other recently published data [42], demonstrate that *Arabidopsis* and a number of other plants have to be considered as true hosts for *Salmonella*. Inoculation experiments suggest that in the cases of *Medicago truncatula* and *Arabidopsis*, *Salmonella* is able to recognize plants as a suitable host and actively enters root tissues (Fig. 1b and [42]). Furthermore, when infiltrated into leaves, *Salmonella* are also able to proliferate in this environment (Fig. 1a and [28]). Most reports so far suggested the presence of *Salmonella* in the apoplast (the interspace between cells) [5], or in the form of biofilms, as shown for parsley [43]. Using GFP-marked bacteria, we show here that, *Salmonella* also inhabits the cytoplasmic compartment (Fig. 1e–h). Only after three hours post inoculation *Salmonella* were present inside root hairs, and 17 hours later also non-root hair rhizodermal cells were infected. By confocal microscopy, bacteria were observed to be moving in the cytoplasm. It is presently unclear whether *Salmonella* are present in endocytotic vesicles comparable to the SCVs in animal host cells. Further studies are also necessary to clarify the exact mechanism how *Salmonella* can penetrate through cell walls and enter into plant cells. Our present findings may however provide an explanation for the common *S. typhimurium* infection after consuming raw, contaminated fruits or vegetables, even after washing or surface sterilization. As shown by our investigation, bacteria present inside plant tissues and/or inside plant cells are resistant to these kinds of treatments and other precautions are required to fulfil current food safety standards.

### Importance of the JA-dependent signaling pathway for defence gene induction

The first event in plant-pathogen interaction is recognition of the pathogen by the plant. Although a number of PAMPs were already identified, only a few receptors have so far been identified. FLS2 [20] and EFR [44] (receptors for flg22 and EF-Tu, respectively) are closely related LRR receptor kinases from the LRR-XII subfamily of receptor-like kinases. In the presence of the respective elicitors, both receptors trigger the activation of downstream kinases and defence responses. A nonproteinaceous binding site for harpinPsph PAMP was identified in tobacco plasma membranes that is required for triggering MAP kinase activation [9]. Activation of MAPK cascades is an essential step to induce defence reactions in response to pathogen attack. Several MAPKs are activated by different plant pathogenic bacteria as well as by treatment with several PAMPs [7], [18], [19], [44]. *Salmonella* attack of *Arabidopsis* plants also results in the activation of MPK3 and MPK6 with a similar kinetics (Fig. 1i), showing a transient activation maximum at 15 min. Since MPK3 and MPK6 are implicated in various pathways, the respective signalling complexes rather than the MAP kinases themselves, are thought to provide the necessary signalling specificity. Since a recent report reveals the MAPK kinase MKK3 to be involved in activating MPK6 in response to JA [25], we investigated a possible involvement of this MAPKK in triggering MPK6 activation in response to *Salmonella* infection. MPK6 activation was totally compromised when *mkk3* mutants were treated with *Salmonella*, revealing that Salmonella-induced activation is mediated by MKK3 (Fig. 6a). In agreement with a role of MKK3 and MPK6 in the defence response against *Salmonella*, we recently found that MKK3 is also involved in defence against bacterial and fungal pathogens and becomes activated by reactive oxygen species [26]. Although MPK6 can also be activated by other MAPKKs besides MKK3 [7], [45], our observation that MPK6 could not be activated to any extent in mkk3 mutant plants speaks against the involvement of other MAPKKs in *Salmonella*-induced activation of the MPK6 pathway. The role of MPK6 is also underscored by the fact that *mpk6* mutant plants were significantly less resistant to *Salmonella* attack, allowing infection to occur significantly faster and the development of higher *Salmonella* levels inside *Arabidopsis* plant tissues (Fig. 6b–c).

Arabidopsis infected by *Salmonella* initiates transcription of a number of defence genes, including the antifungal defensin gene *PDF1.2* [32] and the *PR2* and *PR4* pathogenesis related genes (Fig. 1j, Fig. 4). The transcription of these genes is generally upregulated in response to pathogens as well as by JA and ET [33]. The well-studied SA-regulated *PR1* gene was also upregulated when plants came into contact with *Salmonella* (Fig. 1j). Together, these data indicate that *Salmonella* attack induces in *Arabidopsis* a complex defence response similar to that observed upon attack by other typical plant pathogens [12].

In a simplified view, defence reactions of plants can be divided into SA- and JA/ET-dependent immune responses. SA-dependent responses (further subdivided into NPR1-dependent and NPR1-independent reactions) are important in defence against biotrophic pathogens [13] whereas JA and ET are mainly involved in responses against herbivores and necrotrophic pathogens [16]. We used *Arabidopsis* plants blocked in these three signalling pathways (*NahG*, *coi1–16* and *ein2-1*) in order to determine which if any of these pathways is relevant for resistance against infection by *Salmonella*. The *coi1–16* mutant is defective in an F-box protein required for degradation of repressors of JA-responsive genes [38], [39], and is highly susceptible to *Salmonella* attack (Fig. 5d). The *Arabidopsis coi1–16* mutants were not able to induce activation of MPK6 kinase, nor transcription of any of the tested pathogenesis-related marker genes (Fig. 5f–h), indicating that the JA signaling pathway is absolutely required to induce downstream defence reactions against *Salmonella*. In contrast to Iniquez [28] who reported that *NahG* plants are more susceptible to *Salmonella,* we found slightly enhanced proliferation rates (Fig. 5e), but this may be due to different experimental procedures. Finally, ET was also identified to play a role in Arabidopsis defence against Salmonella. Although the ET signalling *ein2-1* mutant plants showed delayed expression of defence genes that was correlated with enhanced proliferation rates similar to those observed in *coi1–16* mutants, *Salmonella* infected *ein2-1* plants did not produce enhanced disease phenotypes (Fig. 5b). However, this phenomenon does not seem to be a particularity of the interaction between *Salmonella* with *Arabidopsis* plants. Upon infection with pathogenic *Pseudomonas syringae*, *ein2-1* mutant plants showed similar bacterial growth rates as wild type Col-0, plants but only minimal chlorophyll loss [46], indicating that marker gene expression and disease phenotype are not necessarily causally related.

Overall our findings give rise to the following human health considerations. The present work provides an explanation why commonly used practices like washing or surface sterilization of *Salmonella-*infected plants fail to prevent human infection, suggesting that current safety regulations should undergo a serious reconsideration. Alternative strategies to ensure food safety might be achieved by controlling the quality of organic fertilizers and water for irrigation. Moreover, breeding crops and fruits for improved defence signalling could also help to prevent *Salmonella* infection already at the plant level. If plants can be infected with *Salmonella* and thus have be considered as intermediate hosts to spread human diseases, then other human endopathogens could possibly use similar strategies. We therefore also suggest to investigate the potential of other human and animal endopathogenic bacteria to use plants as alternative hosts.

## Materials and Methods

### Plants and bacterial strains

*Arabidopsis thaliana* plants were grown on half MS medium, pH 5.4, 0.7% w/v agar at 22°C under 16 h light conditions (60 µM m^−2^s^−1^ light) or in soil at 22°C under 16 h light conditions. *Escherichia coli* XL1blue (host for cloning experiments) and *S. typhimurium* LB5010 (for generation of recombinant strains), 14028s (wild type), were cultured in LB medium aerobically at 37°C with antibiotic selection, when required.

### GFP marked bacteria

The *gfpmut2* [29] gene encoding green fluorescent protein (forward: ttgaattcgttaactaactaattaatttaagaaggagatatgag, reverse: aaaaaaaagcttccgtctggacatttatttgtatag) was cloned downstream of the constitutive *rpsM* promoter (forward: tctagagaaaggctacggccgttaattgg, reverse: gttaacgccaggatggctttagaacggg) and upstream of *rrnB-*based transcriptional terminators in a high-copy number replicating vector pEC75 (composed of a pUC19-based origin of replication and the pUC19-ampicillin resistance gene). The GFP plasmid was introduced in *S. typhimurium* 14028s. Constitutive expression of GFP in the recombinant strain throughout the growth *in vitro* was verified using Zeiss LSM 510 META confocal microscope.

### Infection of plants with *S. typhimurium*

Bacteria used in infection experiments were grown to late-logarithmic phase. All infections were performed using a bacterial solution with a density of 3×10^8^ cfu/ml. Plants were either cultivated in half MS medium without sucrose and inoculated with bacteria (inoculation experiments) or vacuum-infiltrated with a bacterial solution in 10 mM MgCl_2_ (infiltration experiments). At specific times after infection, plants were surface sterilized in 50 mM PBS pH 7.4 supplemented with 1% v/v bleach, 0.1% w/v SDS and 0.2% v/v Tween20 for 2 min and washed 4 times (2 min each) in H_2_O. Seedlings (inoculation experiments) or 0.7 cm^3^ excised leaf discs (infiltration experiments) were homogenized in 20% glycerol in 10 mM MgCl_2_, and appropriate dilutions of homogenates were plated on selective LB agar media in triplicates. The cfu number was calculated per mg fresh weight per seedling or per leaf disc

### Kinase assays

Kinase activity assays were performed on extracts from 14 days old seedlings treated with *Salmonella* for 0, 15, 30 or 60 min. Cleared cell extracts with equal amounts of proteins were subjected for 2 h immunoprecipitation with 2 µl MPK specific antibodies and 25 µl protein A-Sepharose beads. The kinase reactions of immunoprecipitated proteins were performed at 24°C for 30 min in 15 µl kinase buffer containing 10 µg myelin basic protein (MBP), 0.1 mM ATP and 2 µCi γ^32^P-ATP. Phosphorylation levels of MBP were analyzed with a Phosphorimager.

### Quantitative RT-PCR

Transcript levels were analyzed using the Eppendorf system (*realplex* and RealMaster Mix). 5 µg of total RNA extract were used for reverse transcription. PCRs were run at 95°C, 15 sec: denaturation; 60°C, 15 sec: annealing and 68°C, 15 sec: elongation, for 40 cycles. RNA concentrations were normalized using transcript levels of *clathrin*, *ubiquitin4* and *actin2* genes, and values were plotted as a function of non-infected control samples.

## Supporting Information

Figure S1 *Salmonella* cannot spread between different *Arabidopsis* organs. Single leaves from soil grown 3 weeks old *A. thaliana* wild type Col-0 were infiltrated with a bacterial solution. The plants were cultivated for two additional weeks and cfu of internal bacteria in both infiltrated and non-infiltrated leaves were determined. *S. typhimurium* 14028s was not detected in the non-infiltrated organs. In contrast, bacteria were still present in infiltrated leaves.
(0.05 MB PDF)

Figure S2 *Salmonella* are present in newly formed *Arabidopsis* leaves even one month after infiltration. a–b, *A. thaliana* wild type Col-0 plants were infiltrated with *S. typhimurium* 14028s strain and incubated for an additional month in control growing conditions. Infiltrated leaves died within 5 days (arrowheads in b), however, newly formed leaves were present (arrows in b). cfu number was calculated in discs excited from those leaves two days after infiltration or from newly developed leaves one month after infiltration (a). As control non-infiltrated plants were used.
(0.34 MB PDF)

Figure S3 Z-stacking through an *Arabidopsis* protoplast infected with GFP-marked *Salmonella* cells. One µm optical sections of two infected protoplasts (a–b) were taken using a LSM 510 META confocal microscope and reassembled with the Zeiss LSM Software. 488 nm excitation and 505–530 nm emission filters were used.
(0.45 MB PDF)
