# Supplementary figures and images for: The Dark Side of the Salad: Salmonella typhimurium Overcomes the Innate Immune Response of Arabidopsis thaliana and Shows an Endopathogenic Lifestyle
Source: PLoS One. 2008 May 28;3(5):e2279. doi: 10.1371/journal.pone.0002279 (PMC2386236; doi:10.1371/journal.pone.0002279)

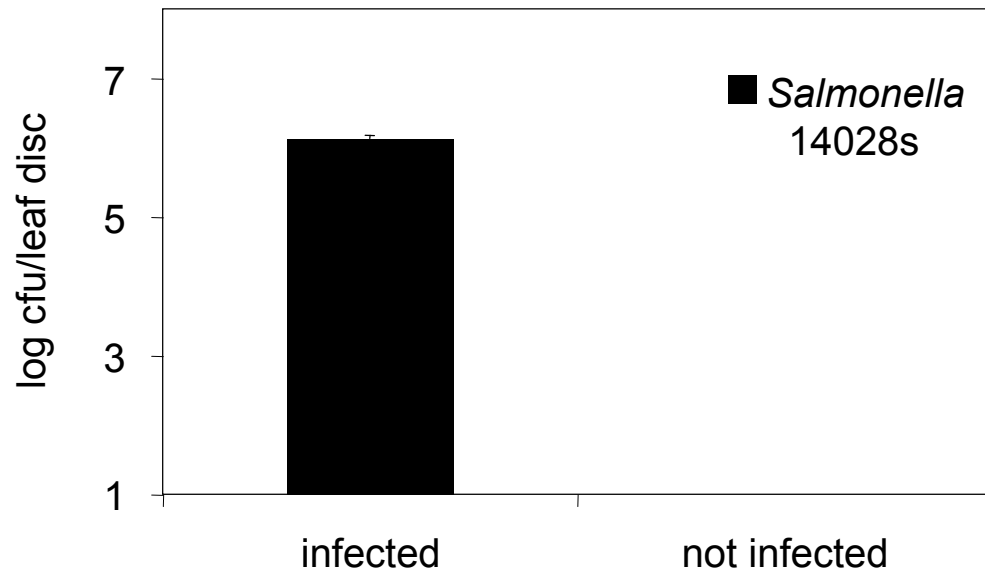

Schikora et al., Figure S1.

Supplement: Figure S1 — Salmonella cannot spread between different Arabidopsis organs. Single leaves from soil grown 3 weeks old A. thaliana wild type Col-0 were infiltrated with a bacterial solution. The plants were cultivated for two additional weeks and cfu of internal bacteria in both infiltrated and non-infiltrated leaves were determined. S. typhimurium 14028s was not detected in the non-infiltrated organs. In contrast, bacteria were still present in infiltrated leaves. (0.05 MB PDF) [file pone.0002279.s001.pdf]

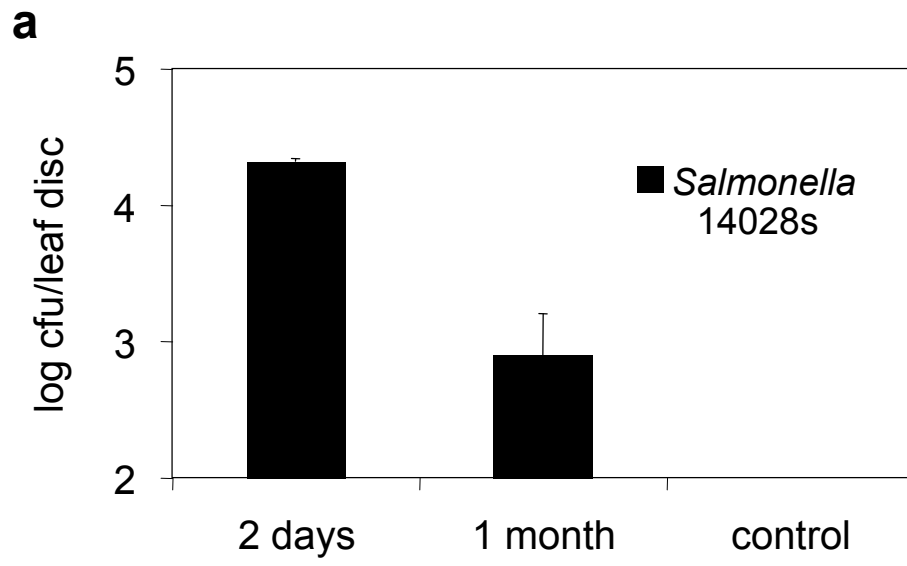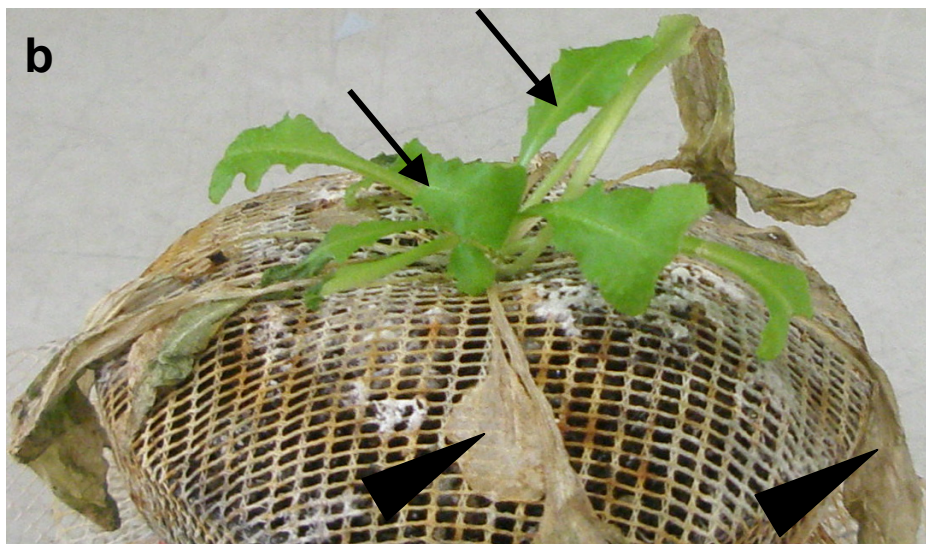

Schikora et al., Figure S2.

Supplement: Figure S2 — Salmonella are present in newly formed Arabidopsis leaves even one month after infiltration. a–b, A. thaliana wild type Col-0 plants were infiltrated with S. typhimurium 14028s strain and incubated for an additional month in control growing conditions. Infiltrated leaves died within 5 days (arrowheads in b), however, newly formed leaves were present (arrows in b). cfu number was calculated in discs excited from those leaves two days after infiltration or from newly developed leaves one month after infiltration (a). As control non-infiltrated plants were used. (0.34 MB PDF) [file pone.0002279.s002.pdf]

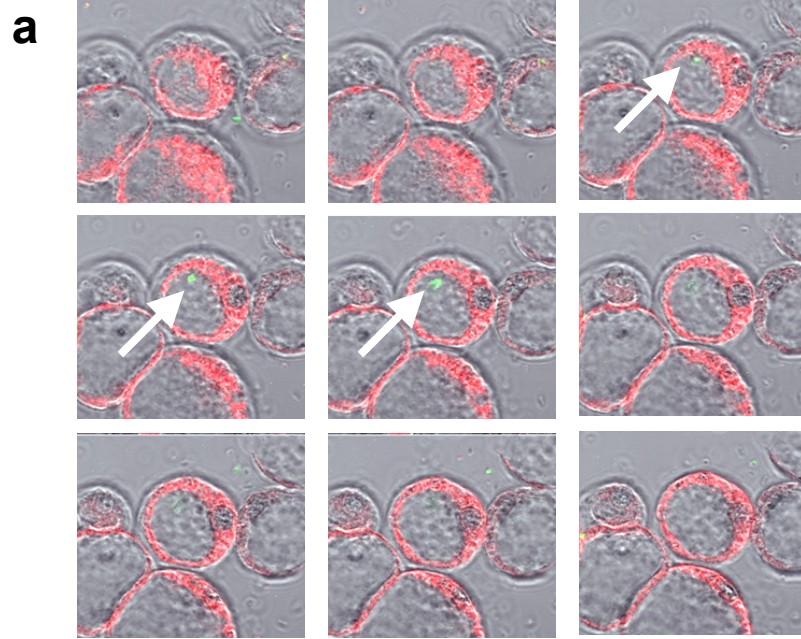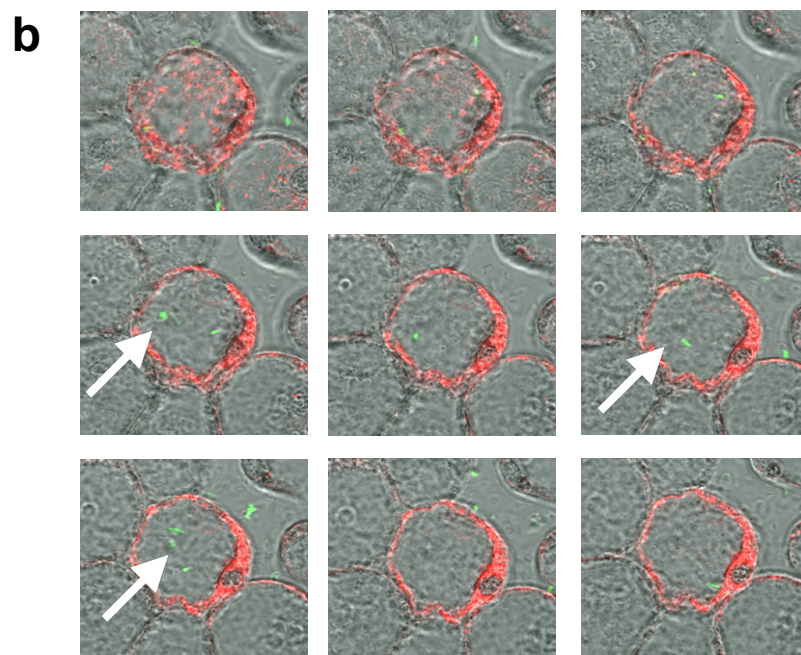

Schikora et al., Figure S3.

Supplement: Figure S3 — Z-stacking through an Arabidopsis protoplast infected with GFP-marked Salmonella cells. One µm optical sections of two infected protoplasts (a–b) were taken using a LSM 510 META confocal microscope and reassembled with the Zeiss LSM Software. 488 nm excitation and 505–530 nm emission filters were used. (0.45 MB PDF) [file pone.0002279.s003.pdf]
